# Understanding geopolitical determinants of health

**DOI:** 10.2471/BLT.20.254904

**Published:** 2021-01-07

**Authors:** Albert Persaud, Dinesh Bhugra, Koravangattu Valsraj, Vishal Bhavsar

**Affiliations:** a The Centre for Applied Research and Evaluation-International Foundation, MHA MacIntyre Hudson, Victoria Court 17-21 Ashford Road, Maidstone, Kent ME14 5DA, England.; b Centre for Affective Disorders, Kings College, London, England.; c South London and Maudsley NHS Foundation Trust, London, England.; d Department of Health Services and Population Research, Kings College, London, England.

Understanding the reasons for health inequality is important to guide policy on global health. One of the approaches to generate evidence to guide such policy is that of the social determinants of health. According to this framework, population health, employment, job insecurity, transport, poverty and housing are key factors of health status and health inequalities in populations.^1^ Comprehensive reviews have been published on the role of commercial and corporate factors in shaping the context of health behaviours (such as smoking and consuming food products that are high in sugar content), the health impact of these factors and related regulations.^2^ A study has also drawn attention to the way in which diverse social contexts over the life course affect health outcomes, and identifies these contexts as the ecosocial determinants of health status.^3^

However, health is also shaped by geopolitical determinants – that is, determinants related to governments, geographies, policies, and the interests of countries and the relationship between them.^4^ Geopolitical determinants are distinct from other health determinants in that they are explicitly reflected in entities such as regions and continental geographies, and proximity and/or distance from neighbours. Geopolitical factors represent a system of relationships among assets and processes that link communities at higher levels of organization than that of the community, neighbourhood or state. Here we argue that understanding these geopolitical determinants can help to advance evidence, advocacy and ultimately policy action to improve global health.^5^

A focus on geopolitical determinants allows the understanding of individual health outcomes as products of national policies at the local and/or regional levels. Such focus may also be helpful in recognizing that these policies are influenced by geographical factors, political leadership (or its absence), relations with neighbouring states and resource distribution.

For example, responses to the coronavirus disease 2019 (COVID-19) pandemic have revealed how different ways of counting deaths and reporting health outcomes and health determinants vary between countries, and how these differences influence the availability, scope and impact of health data and surveillance within countries.

Theoretical approaches to understanding health that do not consider geopolitical perspectives limit the ability to understand the role of local distribution of wealth, poverty, hunger and other such factors, in the structural and geopolitical conditions that shape which health data are collected. Social determinants frameworks do not yet account for the impact of democratic structures or cross-national economic, social and political trends in shaping health. In this regard, the theory of social determinants may be considered an incomplete conceptualization of the distribution of socioeconomic power. This theory does not sufficiently incorporate the historical and geographical patterns of political influence that shape the experience of health care. An empirical approach to conceptualize these trends based on the experiences and perspectives of stakeholders can help to identify new levers for advocacy and practical change, including in the way data are used to support public health improvement.

Geopolitical understandings can give health determinants an additional perspective that might be otherwise overlooked. For example, migration is a key determinant of individual mental health.^6^ Globally, migration flows are shaped by geopolitical processes such as war, famine, colonization, climate change or oppression of minorities. Migrants’ mental health status, which can be assessed from local rates of mental illness and health-care use in migrant groups, is therefore geopolitically determined. While the state of being a migrant intersects with conventional social determinants, including employment, education, life adversity and social support, ignoring the geopolitical dimension of health leads to downplaying the cultural experience of migration and of minority status, which drives chronic stress and affects health. Accordingly, conceptualizing migration flows and migration policy response as geopolitical determinants can contribute to identifying levers for intervening on migration policy to improve health.

Policy-makers’ lack of a sufficiently geopolitical perspective has resulted in limited incorporation of the work of public health researchers and practitioners in this field into public health policies. Geopolitical perspectives on health include the determination of outcomes and collection of health-relevant data such as income, crime, adversity, health surveillance and screening, in addition to the current COVID-19 testing as part of test and trace programmes. These factors transcend national boundaries (and therefore overlap with, but also go beyond national political dynamics) and rest on cross-country comparison. However, these factors are overshadowed by approaches that focus on countries as entirely separate entities. Observable patterns in data on health determinants (such as income, race and employment) are shaped by the ways those data are collected. These data are inherently geopolitical, as reflected in the discussion about ownership and control of the widening 5G telecommunication networks in the United Kingdom of Great Britain and Northern Ireland, the role of China and Huawei in this next-generation wireless technology, and the complex controversy over the ownership and delivery of the United Kingdom COVID-19 contact tracing app. These discussions bring to the fore the importance of understanding state information gathering and/or surveillance mechanisms, and their relationship to security and wider geopolitically relevant policy, as ease of access to big data on health-related metrics has become crucial.

The impact of democratic enfranchisement on health is well recognized in epidemiological research. However, systematic examination of the health impact of advancing state-level populism is still lacking.^7^ An equivalent aspect of populism is the apparently escalating public antipathy towards the concentration of wealth – because wealth tends to determine better health outcomes.^8^ Existing political analyses of health have a predominantly state-based perspective that may not fully address the complexity, porosity and profound individual and societal implications on health of advancing populist political hegemonies around the world. This gap includes circumstances where health challenges, such as infectious outbreaks, in turn shape the geopolitical dynamics that link countries together; as such, the relation between geopolitical factors and health can be bidirectional.

Effective advocacy requires understanding the complex contexts within which policy decisions are made, including the interrelated experiences of agency, risks and benefits among policy-makers. Theoretical frameworks that include geopolitical factors can help understand the links between policy action (for instance through advocacy, research investment and data collection and/or surveillance), education, merging of geopolitical factors such as war, climate change, immigration, health-care policy and health outcomes.

Violence is a key health determinant; therefore, examining health determinants within a geopolitical context allows the identification of state violence, regional violence and the interrelated use of physical and sexual interpersonal violence as key indicators of health system strengthening. Preventing and responding to violent conflict between and within countries is therefore linked to health policy and highlights the importance of violence reduction for public health and public mental health. Thus, geopolitical determinants demonstrate the importance of rational geopolitical understanding and acting on the response of health systems to violence.^9^

More work is needed to describe and quantify geopolitical determinants and implement this understanding through policy and transparent funding that considers geopolitical determinants of health. Limited evidence is available to inform how foreign aid resources should be most effectively and equitably allocated. A recent ecological study examined the relationship between the level of aid from the United States of America across countries and its national security threat level. As well as an overall reduction in state aid from 2009 to 2016, higher threat levels were accompanied by greater declines in spending.^10^ Therefore, evidence exists that global security concerns play an important role in foreign aid (which in turn is relevant for health in low- and middle-income countries). However, such an analysis does not account for the importance of state fragility and vulnerability, or the ways in which the drivers of insecurity, such as regional violence, corruption, political instability and disenfranchisement, drive poor health outcomes.^11^

Hence, renewed attention to factors that shape state vulnerabilities across a range of geopolitical domains may allow for fairer distribution of aid resources. Reforming global governance for health and improving the capacity of low- and middle-income countries to transparently negotiate and safeguard their interests is therefore necessary. We have previously proposed the country-level Compassion, Assertive action, Pragmatism and Evidence Vulnerability Index, which could guide such discussions and drive greater health equity.^12^ Health researchers should consider the geopolitical context within which individuals experience exposure to geopolitical determinants, and within which health outcomes are defined and captured. Health practitioners should consider incorporating the geopolitical context to the design and delivery of individual health care into training materials. A geopolitical perspective can also help policy advocacy identify appropriate levers for change, including in law and surveillance strategies.

Much previous theorizing on the social determinants of health emanates from high-income countries and has not taken stock of ongoing changes in the distribution of political and socioeconomic influence between countries. We think that the COVID-19 pandemic, against the backdrop of rapid economic growth in Brazil, China, India and the Russian Federation, calls for consideration of the geopolitical determinants of health. Focusing only on social determinants of health may not sufficiently capture the complex contexts in which health policy decisions are made. In 2020, many policy-makers and health organizations consider public health to be at the heart of economic and social policy thinking. Geopolitical perspectives can be helpful to make more comprehensive and better-informed decisions on health. Such an approach could improve the reach of public health evidence and the way it is used to shape advocacy. Understanding health challenges and policy responses as geopolitically shaped could help identify unintended consequences of not factoring in geopolitical determinants of health in countries, by allowing the inclusion of policy experiences from other settings.
